# A water droplet-cleaning of a dusty hydrophobic surface: influence of dust layer thickness on droplet dynamics

**DOI:** 10.1038/s41598-020-71743-y

**Published:** 2020-09-08

**Authors:** Ghassan Hassan, Bekir S. Yilbas, Saeed Bahatab, Abdullah Al-Sharafi, Hussain Al-Qahtani

**Affiliations:** 1 Department of Mechanical Engineering, King Fahd University of Petroleum and Minerals (KFUPM), Dhahran, 31261 Saudi Arabia; 2 K.A.CARE Energy Research and Innovation Center, Dhahran, Saudi Arabia; 3 Center of Research Excellence in Renewable Energy (CoRE-RE), King Fahd University of Petroleum and Minerals (KFUPM), Dhahran, 31261 Saudi Arabia

**Keywords:** Energy science and technology, Engineering

## Abstract

Water droplet cleaning of a dusty hydrophobic surface is examined. Environmental dust are used in the experiments and cloaking velocity of a dust layer by a droplet fluid is measured and hemi-wicking conditions for the dust layer are analyzed adopting the pores media wick structure approach. A droplet motion on dusty and inclined hydrophobic surface is analyzed using a high speed digital imaging system. Influences of dust layer thickness, droplet volume, and surface inclination angle on the mechanisms of dust removal by a rolling droplet are evaluated. The findings revealed that dust cloaking velocity decreases exponentially with time. The droplet fluid can cloak the dust layer during its transition on the dusty surface. The transition period of droplet wetted length on the dusty surface remains longer than the cloaking time of the dust layer by the droplet fluid. Translational velocity of rolling droplet is affected by the dust layer thickness, which becomes apparent for small volume droplets. Small volume droplet (20 µL) terminates on the thick dust layer (150 µm) at low surface inclination angle (1°). The quantity of dust picked up by the rolling droplet increases as the surface inclination angle increases. The amount of dust residues remaining on the rolling droplet path is relatively larger for the thick dust layer (150 µm) as compared to its counterpart of thin dust layer (50 µm).

## Introduction

Environmental dust settles on surfaces and alters surface optical characteristics while influencing performance of a renewable energy devices such as photovoltaics, which becomes significant over the time^1^. This is because of the composition of the environmental dust, which is consisted of various elements and oxides while lowering the UV visual transmittance of solar radiation^6^. Surface cleaning of such devices becomes essential for effective and sustainable operations. Although several methods have been proposed for surface cleaning^2,3^, a water droplet cleaning of dusty surfaces is one of the cost-effective cleaning processes. For the droplet cleaning process, the droplet rolling/sliding on surfaces is necessary; hence, a hydrophobic state on the surface becomes essential. Achieving sustainable hydrophobic state with low contact angle hysteresis in outdoor environments is challenging. The harsh environmental conditions, such as high humidity and extremely hot temperature conditions, influence the surface characteristics. In this case, alkaline and alkaline earth compounds of dust dissolve in water condensate on the surface in humid air ambient. This gives rise to chemically active solution formed on the surface while damaging the surface^4^. Hence, sustainable hydrophobic wetting state on surfaces becomes desirable for long term operation in outdoor conditions. Surface coating with functionalized silica nano-units offers durable wetting state in outdoor environments.

On the other hand, droplet motion on hydrophobic surface becomes critical for cleaning process. Increasing droplet size results in bulging of droplet while causing enlargement of the droplet wetted diameter on the surface. This effects the droplet pinning, due to interfacial shear, and slows down the droplet speed on the surface. The droplets with small rolling/sliding velocities puddle irregularly on the surface and the dust removed area shows striations like appearances^5^. In addition, dust residues are observed on the surface. In general, the dust is cloaked by the droplet fluid during rolling/sliding motion on the surface and the droplet picks up the dust during rolling/sliding. However, the dust particles with sharp edges can anchor on the surface and they are difficult to pick up by the droplet. The low surface energy dust particles are unable to be cloaked by the droplet fluid and mostly they remain as residues on the surface while adversely affecting the cleaning performance^6^. In addition, the dust thickness influences the droplet fluid cloaking and the amount of dust picked up by the droplet may reduce with increasing dust thickness on the surface. Consequently, investigation of the dust cloaking mechanism and the effect of dust thickness on the surface cleaning process becomes essential.

In the case of high contact angle hysteresis of the surfaces, the external affects, such as electrodynamics influence, can also be used to mobile the droplet on the hydrophobic surfaces^7^. The electrodynamics affect can be utilized for self-cleaning applications of surfaces by rolling/sliding droplets^8^. In addition, electrostatic charges can be used to repel the dust particles from the surfaces^9^; however, depending on the electrostatic force generated on the surface, the particles repelling creates dust cleaning effects on surfaces. Introducing electrostatic repelling force on the hydrophobic surface creates more effective dust removal than that of the hydrophilic surfaces. This behavior is attributed to the reduced adhesion between the dust and the hydrophobic surface^9^. Moreover, the lateral force related to the adhesion under surface tension plays a major role in droplet behavior on the surface. The adhesion energy at the droplet-surface interface determines the dynamics of the droplet motion^10^. The surface structure influences considerably the lateral force balance between the droplet and the surface^11^; hence, hierarchical texture distribution becomes favorable for steady droplet behavior on the surface. Introducing external influence can, possibly, minimizes droplet adhesion on the hydrophobic surfaces. Applying external vibrations^12^ or generating thermocapillary forces in the droplet fluid alters the droplet dynamics on the hydrophobic surface, i.e. small temperature difference (ΔT ~ 4 °C) between the fluid and the surface can generate Marangoni dominated flow in a droplet^13^.

One of the mechanisms governing dust removal by a rolling droplet is related to cloaking of dust by droplet fluid^5^. Depending on a droplet transition on a hydrophobic surface, duration of droplet fluid-dust interaction changes. In some cases, droplet transition time may become similar order of magnitude of interaction time and a dust layer may not be cloaked by rolling/sliding droplet. Since settled dust composes of various size and shapes of the particles, the dust acts like a porous layer on the surface. Liquid spreading and wicking phenomena can change on the dusty layer depending on the layer structure on the hydrophobic surfaces. Capillary force variation due to composite wick structure has been investigated previously^14–16^. Hydrostatic pressure loss is associated with the flow resistance in the porous-like structure and wetted height and spreading velocity of liquid change with the permeability of the structure. The dynamics of a liquid penetration due to nano-scale hydrophilic capillary structures are involved with multi-physics phenomena and formulation of wicking velocity by using the Washburn’s equation does not provide the exact solution to a hydrophobic capillary phenomena^17^. The dynamic contact angle variation of the surface alters the liquid penetration rate. Hence, the Washburn’s equation fails under the conditions: (i) porous height comparable to few molecular size, and (ii) large values of liquid spreading rate. Nevertheless, the Washburn’s equation provides useful information for porous-like structures with length scale much larger than the molecular sizes. Moreover, microgrooves resembling hydrophobic texture topology changes the heat transfer rates on the surface due to micro-capillary influences. Hence, wicking ability of the surface has significant effect on the heat transfer rates^18^, which becomes apparent as the groove shape changes to rectangular micro-grooves, i.e. heat transfer and evaporation rates further improve^19^.

Dust forms porous-like structures in outdoor environments by time and excessive dust accumulations on the surfaces can limit the efficient dust removal from surfaces by rolling/sliding droplets. The wicking condition pertinent to porous-like structures is presented earlier^14–20^, however, a droplet rolling/sliding on porous structures, due to dust settlement on the hydrophobic surfaces, have not been investigated. In addition, as the accumulated dust height increases, the area of hydrophobic surface exposing to droplet interface becomes small and droplet rolling/sliding characteristics change, i.e. rolling may cease and the dusty surface acts like a hydrophilic behavior due large area coverage of the dust particles on the hydrophobic surface. Consequently, in the present study, the droplet rolling characteristics on inclined dusty hydrophobic surface is investigated and the influence of dust layer thickness on rate of dust picked up by the droplet is examined. Experiments are carried out determining the spreading rate of the droplet fluid on the dust layer and assessing the influence of dust layer thickness on the droplet behavior. Dust layer wicking conditions are also examined using high speed optical and thermal cameras.

## Experimental

Glass samples were hydrophobized through coating by functionalized nano-silica particles. The wetting state of the sample surfaces prior to coating was determined via contact angle measurement and it was found to be hydrophilic with contact angle of about 55°. The contact angle measurements were repeated 5 times at each locations on the surface, ensuring the repeatability of the contact angle data, while adopting the procedure introduced in the early study^20^. Nano-size silica units were synthesized and, later, they were functionalized^21^. During the processing, the mixture of ethanol (14.2 mL), desalinated water (1.2 mL), and ammonium hydroxide (24 mL) was prepared through stirring at 360 rpm for 18 min. Later, diluted tetraethyl orthosilicate (TOES) (1.5 mL TOES in 4 mL ethanol) was included in the stirred mixture and it was left for 20 min. The modifier silane was included in 3:4 molar ratio to the resulting solution and it was, later, stirred magnetically for 12 h. After accomplishing stirring, the solution was centrifuged and reactants are removed by addition of ethanol. A dip coating technique was adopted for coating sample surfaces by the resulting solution. Samples were vacuumed for drying and removing residues through evaporation. The dip coating process gives rise to a uniform coating thickness of 500 ± 30 nm. The coating surfaces were micro-imaged via a scanning electron microscope (SEM, JEOL 6460). Surface characteristics of the coating (roughness and roughness parameter) were evaluated via incorporating an atomic force microscope line scan (AFM, NanoMagnetics Instruments). Hydrophobic state of the sample surfaces was estimated incorporating the contact angle method^20^. The droplet on the dusty hydrophobic surface was monitored using a high speed imaging and recording system (SpeedSense 9040). The camera operated at 10,000 to 5,000 frames-per-second (fps) in the experiments at its full megapixel resolution was 1,280 × 800 with the pixel size of 14 µm × 14 µm. In addition, the dust cloaking velocity was evaluated (SpeedSense 9040).

The dust was gathered around Dammam area in Saudi Arabia over the six months periods. A care was taken during the dust collection; hence, soft brushes were used removing dust from the photovoltaic panel surfaces. Later, collected dust was enclosed in airtight containers. The dust thickness was measured at different locations over six month periods. The thickness varied between 50 to 150 µm and at some locations extreme dust thickness was also observed (> 250 µm) as consistent with the early work^4^. The dust were examined under scanning electron microscope for sizing and shape assessing. Energy dispersive spectroscopic were conducted towards analyzing dust elemental composition. X-Ray diffraction (D8 Advanced diffractometer) with CuKα radiation was utilized evaluating the dust compounds.

## Results and discussion

Effect of dust layer thickness on the droplet behavior is examined and droplet the fluid spreading rate on dust surface is determined. Wicking conditions for dust layer is assessed and the dust cleaning process by rolling droplet is evaluated.

### Hydrophobic surface and dust particles

**Figure 1:**
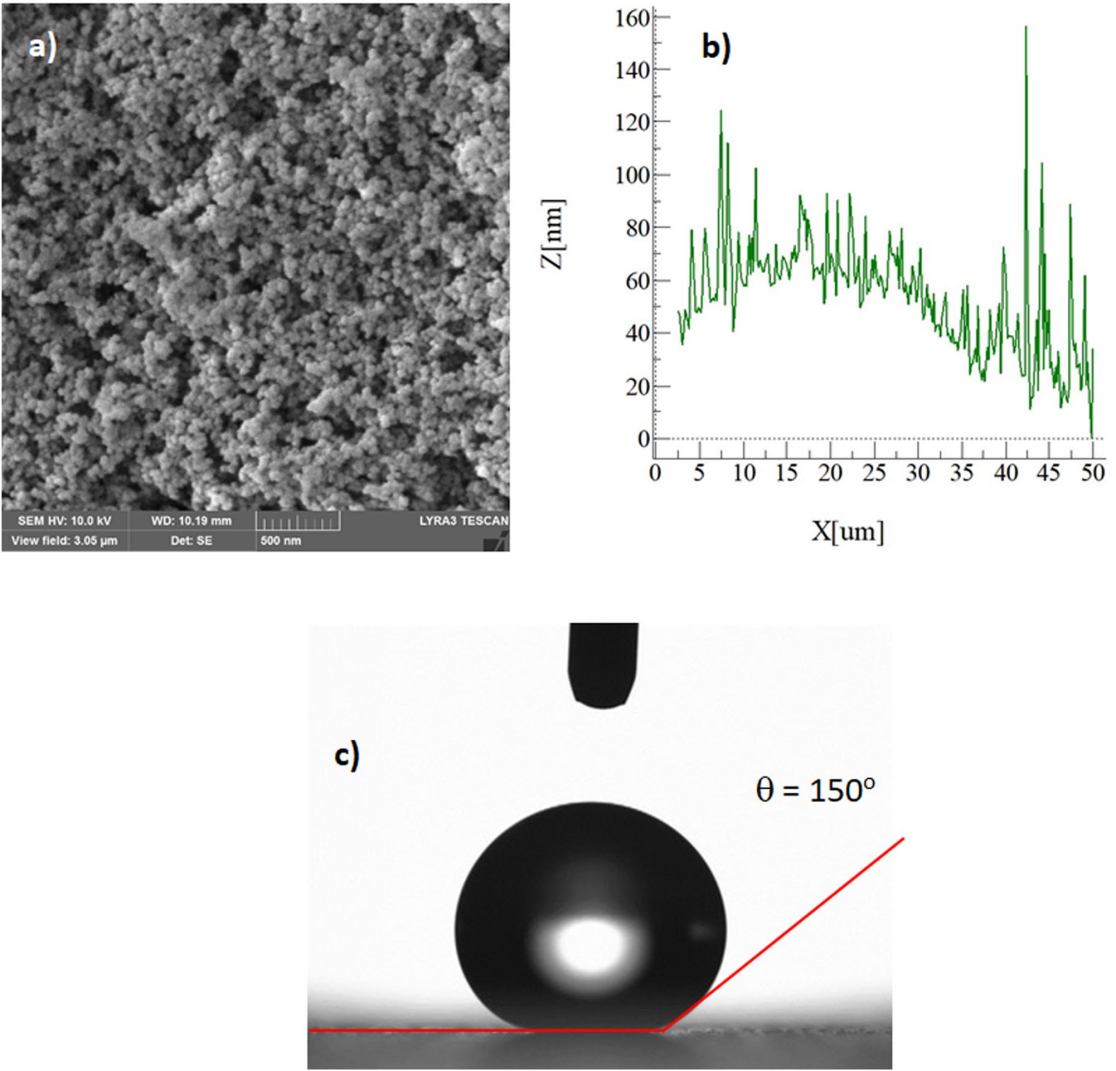
(**a**) SEM micrograph of coated surface, (**b**) AFM line scan on coated surface, and (**c**) droplet contact angle on coated surface.

**Figure 2:**
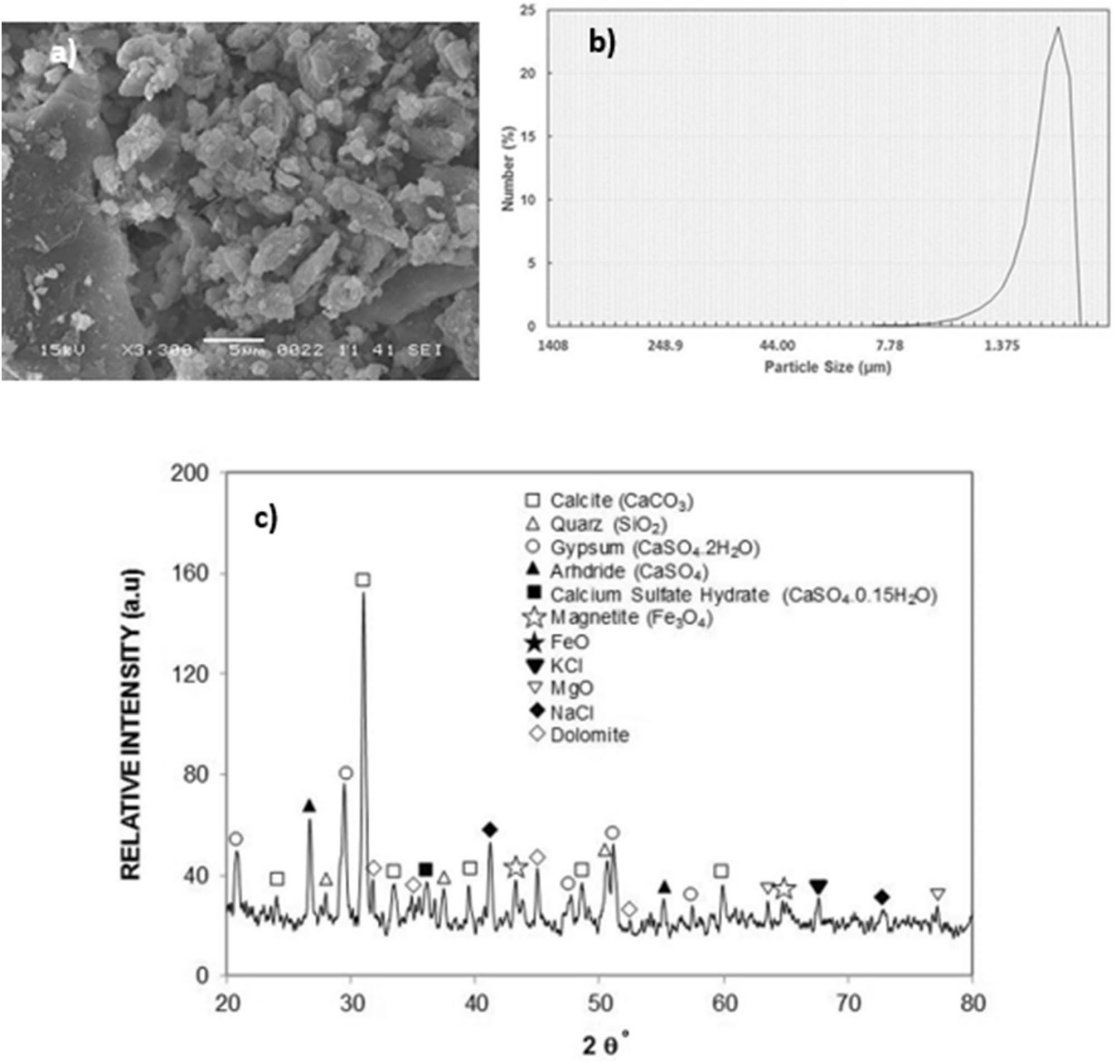
(**a**) SEM micrograph of dust particles. Small particles adhere on large particle and small particles form a cluster, (**b**) size distribution of dust particles, and (**c**) X-ray diffractogram of dust particles.

Figure 1a depicts SEM images of functionalized nano-silica particles coated surface while Fig. 1b shows atomic force microscopy of the surface line scan. The coated surface resembles closely formed nano-size silica units with a nominal size of about 30 nm. Small size porous appearance on the surface (Fig. 1a) is related to agglomeration of nano-silica units, i.e. modifier silane gives rise to the side reactions during condensation on silica particles, which trigger silica particles agglomeration^22^. Agglomerated particles do not form a thick coating layer on the surface. It can be noted that once the particles agglomerate in the coating the coating thickness is expected to increase, which generates a texture profile while altering the wetting state locally. However, from atomic force microscope line scan, the average roughness of the coated surface is about 65 nm (Fig. 1b). Hence, the coating produced has fine-size thickness and the agglomerated particles enable to form air-gaps in the coating (Fig. 1a). In addition, some rippling behavior is noted along the surface line scan, which represents the clustered particles on the surface (Fig. 1b). The roughness parameter of the coating surface is determined using surface topology obtained from the atomic force microscope. The roughness parameter, which corresponds to the ratio of pillars area over the projected area^23^ and it is about 0.49 for coating surface. The state of the wetting of the coating surface is evaluated adopting the contact angle method^20^. In addition, advancing and receding angles of the droplet are also measured and contact angle hysteresis is assessed. Figure 1c depicts droplet image on the coating surface. The contact angle of the coating surface is about 150° ± 2° and hysteresis is about 2° ± 1°. The measurement contact angle and hysteresis is repeated five times at leach location on the surface while incorporating the technique used in the early work^20^. This arrangements ensures the repeatability of the contact angle data. Droplet rolls on the coating surface rather than sliding, i.e. pinning of droplet due to adhesion under the effect of surface tension is significantly small. The free energy of the coated surface is determined via contact angle measurements^24,25^ and it is estimated about 35.51 mJ/m^2^. On the other hand, the dust particles collected are analyzed using scanning electron microscope (SEM), energy dispersive spectroscopy (EDS) and X-ray diffraction (XRD). Figure 2a depicts SEM image of the dust particles while Fig. 2b shows dust particle size distribution. The particles have various sizes and shapes (Fig. 2a,b). Small particles cluster and attaches to large particles (Fig. 2a). The shape of particles can be assessed through introducing a shape factor parameter^4^. The parameter is determined from the estimation of cross-sectional area and dust perimeter (${R}_{Shape}= \frac{4\pi A}{{P}^{2}}$, here *P* represents the dust perimeter and *A* is the cross-sectional area). The shape factor resembles the circularity of a dust particle. SEM micrographs of individual dust particle are used determining the dust particle perimeter and the corresponding dust particle cross-section. However, no strong correlation is observed between the dust size and the shape factor. Nevertheless, the value of shape factor improves with increasing dust size. Hence, it is close to 1 for dust with size ≤ 2 μm, and the median of shape factor becomes 3 for dust with size ≥ 5 μm. Table 1 gives EDS dust data (in wt%). Dust contains Na, K, Si, S, Ca, Fe, O, Mg, and Cl. The existence of Na, K, and Cl in the dust reveals that dust contain salt compounds. Chlorine concentration is higher for large size dust (> 1.2 µm) and Cl concentration does not satisfy the stoichiometric ratio for NaCl; hence, NaCl is present in the dust with nonstoichiometric concentration. X-ray photoelectron spectroscopy (XPS) is carried out towards assessing Na, K, and Cl binding energies. The findings reveal that chlorine covers inorganic chloride, i.e. Cl2p_3/2_ peak appears at 198.9 eV (3a) as consistent with early studies^26,27^. The data obtained from CasaXPS indicate that the concentration ratio (in terms of the mass base) of potassium over chlorine is about 1.62, which is similar to that obtained from the energy dispersive spectroscopy data (Table 1). Moreover, sodium data demonstrate that the XPS peak occurs at 1,072.8 eV for Na1s. Hence, CasaXPS shows that the ratio of sodium concentration over chlorine (in terms of mass percentage) is 2.67, which is similar to that of the energy dispersive spectroscopy data (Table 1). To evaluate the dust compounds, X-ray diffractogram is carried out. Figure 2c depicts X-ray diffractogram of the dust collected, which possess same to the data reported in the early work^5^. Here, the peaks of Na and K are because of salt while sulfur is because of calcium, such as in anhydrite or gypsum components (CaSO_4_). However, Fe is because of hematite (Fe_2_O_3_) in the dust particle. To assess the surface energy of dust, the contact angle technique is adopted, as presented in the early studies^24,25^. It is should be noted that the Washburn technique can be used to assess the surface free energy of porous-like structures, such as dust layer^28^. The Washburn technique^28^ is accommodated measuring the contact angle of three-different fluids (water, glycerol, and ethylene glycol). A glass-tube of 3 mm diameter is for the assessment of the selected liquid contact angle, which are drawn-up in the tube under the capillary force. The mass increase in the tube and the time for this mass increase is related through the Washburn technique, which is: $\frac{{\Delta m}^{2}}{\Delta t}=\frac{c.{\rho }^{2}\gamma cos\theta }{\mu }$, where *Δm* is the mass gain, *Δt* is the time corresponding to mass gain (flow time), *c* is the capillary constant of the dust, *ρ* is the fluid density, *θ* is the contact angle, *µ* is the fluid viscosity. The capillary constant of dust is evaluated using n-hexane as a liquid, which results in zero contact angle (*θ* = 0). Hence, the variation of mass gain square (${\Delta m}^{2}$) with corresponding time ($\Delta t$) provides the contact angle of the fluid. The capillary constant for dust particles is estimated as 5.82 × 10^–16^–6.54 × 10^–16^ m^−5^. However, the variation of particle size influences the capillary constant^29^. Hence, the variation of capillary constant may be associated with dust particle sizes and shapes, which are different. Moreover, to validate the contact angle measurements, dust pellets are prepared in line with early work^29^. In the process, dust particles are lightly compressed to form pellet-like samples. Water, glycerol, and ethylene glycol are used in the experiments. The contact angle determined from Washburn technique slightly differs from that of measured from the dust pellet surface, i.e., the contact angle measured on the pellet surface for water is about 38.2° while the Washburn technique results in 37.4°. Nevertheless, the difference is small. Table 2 gives the Lifshitz-van der Walls components and electron-donor parameters used in the surface free energy assessments^24,25^. The surface free energy measured for the dust pellets is about 112.2 ± 5.2 mJ/m^2^. The experiments are repeated twelve times to secure the accurate measurement of the surface free energy of the dust pellets.

**Table 1:** Elemental composition of dust particles (wt%).

	Si	Ca	Na	S	Mg	K	Fe	Cl	O
Size ≥ 1.2 μm	11.8	8.3	2.2	1.3	2.5	0.8	1.2	0.4	Balance
Size < 1.2 μm	10.2	7.3	2.7	2.5	1.3	1.2	1.1	1.1	Balance
Dust residues	9.5	7.1	1.9	1.3	2.4	0.9	0.9	0.4	Balance

**Table 2:** Lifshitz-van der Walls components and electron-donor parameters used in the simulation^24,25^.

	γ_L_ (mJ/m^2^)	${\gamma }_{L}^{L}$(mJ/m^2^)	${\gamma }_{L}^{+}$(mJ/m^2^)	${\gamma }_{L}^{-}$(mJ/m^2^)
Water	72.8	21.80	25.5	25.5
Glycerol	63.3	33.11	10.74	21.23
Ethylene glycol	48.2	31.09	6.59	11.16

**Figure 3:**
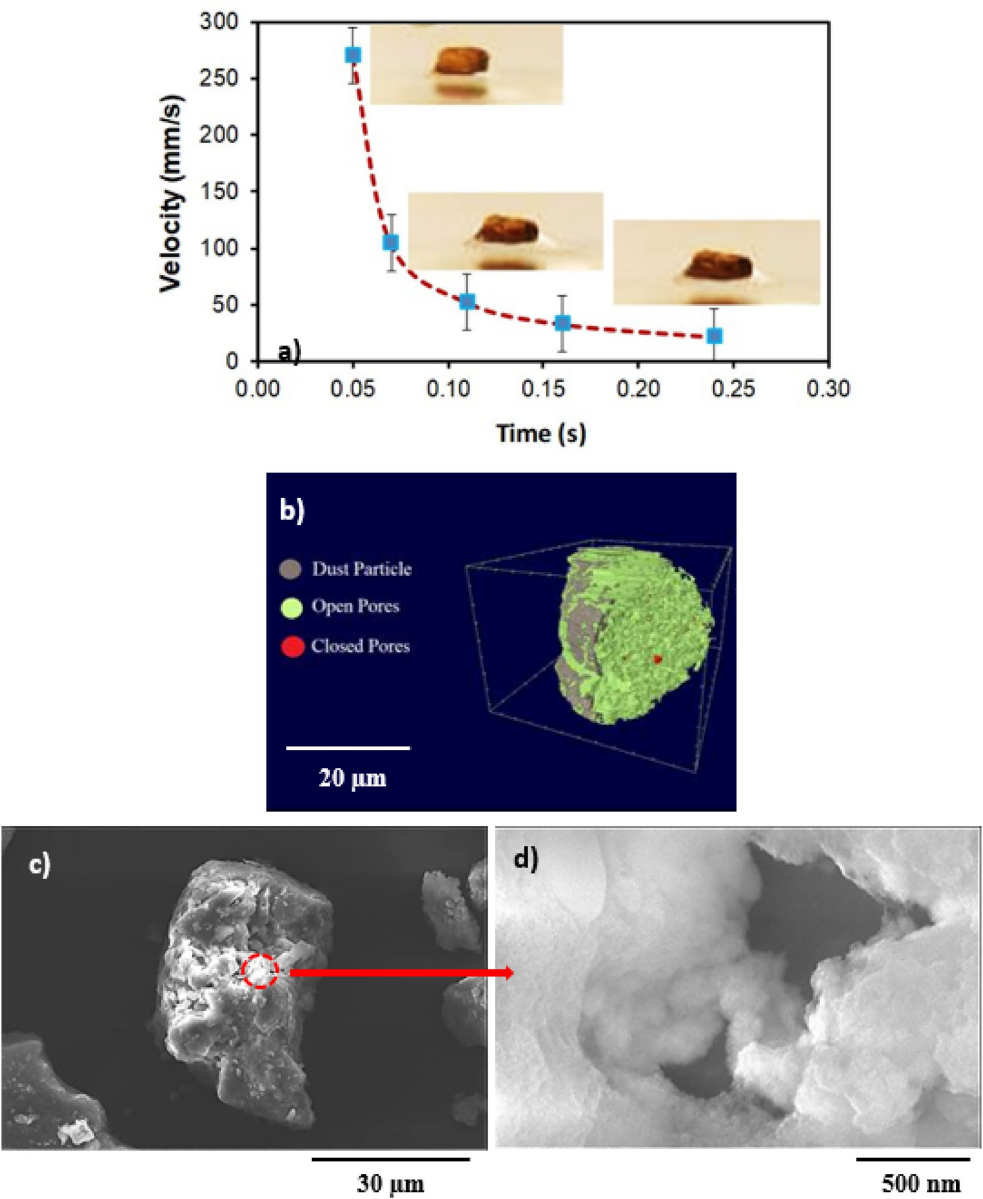
(**a**) Cloaking velocity of water on a dust particle, (**b**) CT scan of a large dust particle, (**c**) SEM micrograph of a large dust particle, and (**d**) SEM micrograph of magnified large particle surface.

**Figure 4:**
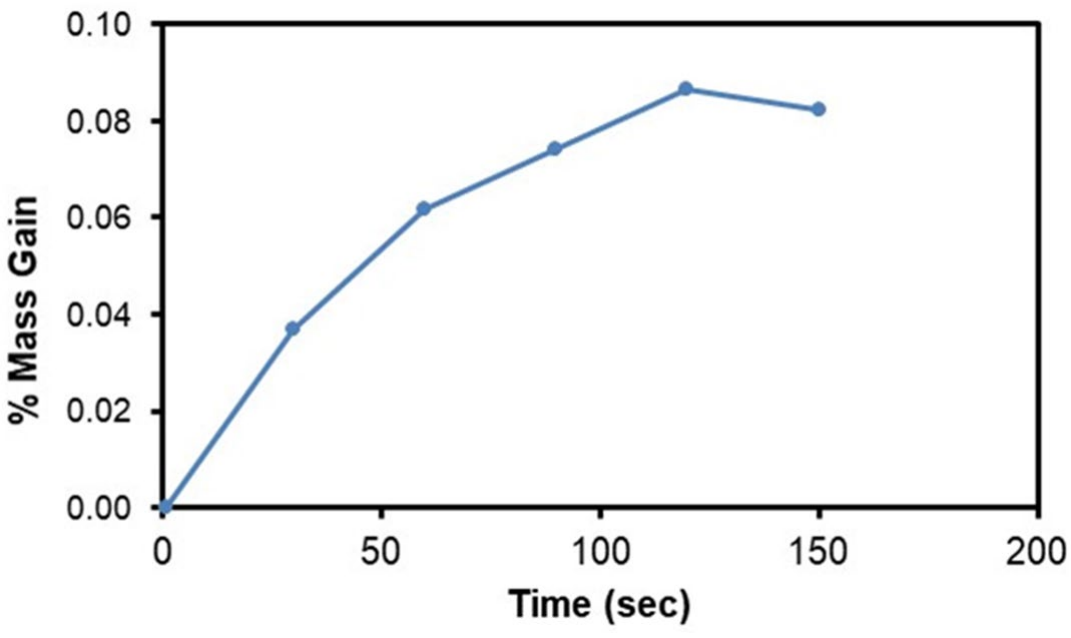
Percentage of weight gain of a dust particle in water after cloaking is completed during 150 s.

**Figure 5:**
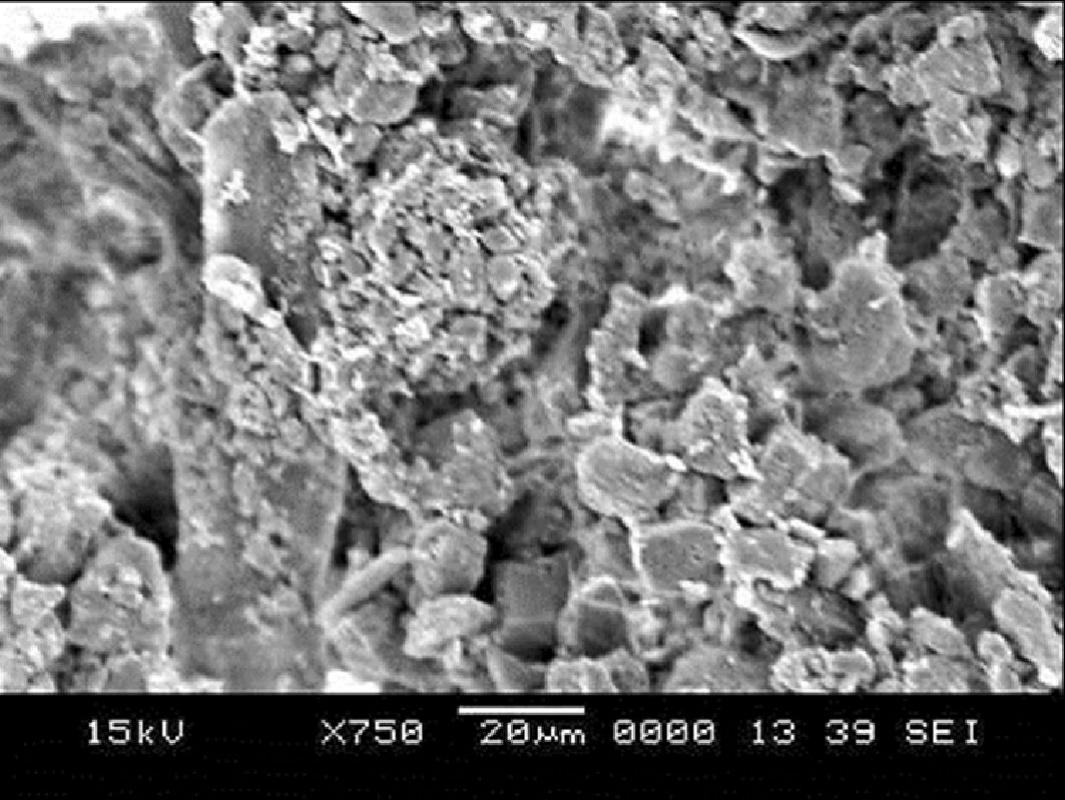
SEM micrograph of dust column cross-section. Large porosity is observed on micrograph.

Droplet liquid wets dust particles during rolling on the dusty hydrophobic surface. This forms a liquid wrapping layer on the dust surface and gradually infusing liquid, which is droplet fluid (water), encapsulates the dust surface. This phenomenon is defined as the liquid cloaking^30^. The droplet liquid spreading on the dust surface satisfies the hemi-wicking criteria^31^, which yields: $S={\gamma }_{s}-{\gamma }_{L}-{\gamma }_{s-L}$, here, *γ*_*s*_ represents dust surface free energy, *γ*_*L*_ is surface tension of droplet liquid, and *γ*_*s-L*_ resembles the interfacial tension at droplet liquid and the dust interface. The interfacial tension at droplet interface can be devised as:${\gamma }_{s-L}={{\gamma }_{s}-\frac{{\gamma }_{L}}{r}cos\theta }_{w}$, ^32^, here *θ*_*w*_ corresponds to contact angle of a droplet fluid on the pellet surface and *r* represents the roughness parameter of the pellet surface. 3-dimensional imaging microscope is used to determine the roughness parameter, which is found to be 0.61. The contact angle *θ*_*w*_ is measured as 37° ± 4°. Therefore, ${\gamma }_{s-L}=18.23$ mJ/m^2^ and inserting in spreading rate (*S*) formula, it yields *S* = 22.27 mJ/m^2^, i.e. *S* > 0. Hence, the droplet fluid (water) spreads over the dust surface. Experiments are carried out to determine the water cloaking velocity on the dust surface. In this case, high speed video recording using the micro-lens was utilized to monitor ridges of water on dust particle surface. The tracking program is used to monitor and temporal variation of the height of water ridges on dust surface via uploading the high speed video recorded. Figure 3a shows the cloaking velocity with time for a single dust particle. The cloaking velocity sharply decays with time and shows almost quasi-steady behavior with progressing time until the particle is totally cloaked. The variation resembles the exponential decay in the form of ~ C.e^-mt^, where C is a constant and m is the parameter which is about 0.25 s^−1^. The exponential decay of the cloaking velocity is also reported in the early work^33^ and it occurs because of the force balance among the gravity, surface tension, interfacial tension, and shear resistance^34^. Energy dissipated due to shear resistance during the cloaking is related to the Ohnesorge number ($Oh={\mu }_{o}/\sqrt{{\rho }_{o}a{\gamma }_{L}}$); here, ${\gamma }_{L}$ is the surface tension and *a* is particle size^35^. Incorporating the dust particle size ≥ 1.2 µm, the Ohnesorge number becomes much less than one (*Oh* ~ 0.057). Hence, the dissipation force due share rate becomes small during the cloaking. On the other hand, the dust particle has open porous structure, which can cause droplet water penetration into the dust particle via porous sites. Figure 3b shows computerized tomography (CT) nano-scan of a dust particle. The size of the dust particle is about 25 µm and it is difficult to measure the pore size less than 0.5 µm from nano-CT scan image (Fig. 3b). This is because of the resolution of nano-CT scan, which is 0.5 µm. However, SEM micrographs are analyzed to assess the average pores size. SEM micrograph of a typical large size dust particle is shown in Fig. 3c while Fig. 3d depicts SEM micrograph of magnified large dust particle surface. The pore size various on dust surface and the averaged pore size is estimated at about 450 nm. The open porous sites covers about 35% of the dust particle. To evaluate liquid (water) penetration into the dust particles via porous sites, experiments are carried out to determine mass gain by the dust particle with time. Since, the transition time of droplet wetting diameter on the dusty hydrophobic surface and cloaking time of droplet fluid are small, which is in the order of fraction of a second, experimental duration for the weight gain of the dust particle is extended in the order of 10 times of the cloaking and the transition times. Figure 4 shows the percentage of the mass gain of the dust particle with time. The mass gain of the dust particle is determined from the ratio of difference between final and initial masses of the dust particle over the initial mass of the dust particle. The initial and final mass of the dust particle is measured using sensitive scale (Thomas Scientific). Since the liquid film is formed on the dust particles after complete cloaking, the final mass of the dust particle is measured for the periods after the cloaking period. The initial mass of the particle is measured onset of complete cloaking. Moreover, the percentage of the mass gain of the dust particle ($\frac{({m}_{cl}-{m}_{dr})}{{m}_{dr}}$, here *m*_*cl*_ is the particle mass after cloaking and *m*_*dr*_ is the mass of dry particle)) during the cloaking is also measured and it is about 0.1728, i.e. almost 17.28% of the mass of the dry dust is increased during cloaking period. In line with Fig. 4, as cloaking period increases further, the mass gain of the particle remains small, which in the about 0.1% after the duration 140 s inside water after the cloaking period. Hence, infusion of the droplet fluid through the porous sites of the dust particle is extremely small. Consequently, the mass gain of the dust particle during the transition time of the droplet wetting area on the dusty hydrophobic surface is almost limited with the mass of the liquid covering the dust surface due to cloaking. Moreover, the dust layer is considered to have porous structure and the wetted height of the liquid in the dust layer is estimated experimentally. In this case, 3 mm diameter the dust columns with 12 mm height are prepared from the dust particles. High speed recoding system is used to monitor the wetted front in the dust column while the column is located on the liquid (water) film. Infusion of the liquid into porous dust column is governed by the capillary pressure and the pressure drop due to fluid weight and porous resistance along the column height. The capillary pressure can be estimated from Laplace Young Equation^36^ as: $\Delta {P}_{Cap}=\frac{2{\gamma }_{L}}{{R}_{eff}}$, here, *γ*_*L*_ represents surface tension of fluid and *R*_*eff*_ is the effective capillary radius. The pressure drop (pressure loss) can be associated with the friction due to pores structure and gravitational influence (hydrostatic) during the liquid infusion. The pressure loss ($\Delta {P}_{loss}$) can be formulated incorporating the friction and hydrostatic influences, which yields^36^: $\Delta {P}_{loss}=\frac{\mu \varepsilon }{K}h\frac{dh}{dt}+\rho gh$, here *µ* is the fluid viscosity, *ε* is the porosity, *K* is permeability, *h* is the wetting fluid height, *ρ* is the fluid density, and *g* is the gravity. SEM micrograph of the cross-section of the dust column (Fig. 5) is used to estimate the porosity. The porosity is determined as the ratio of area covered by pores over the cross-sectional area, which is estimated at about 0.18. Several dust columns are produced from the dust particles to assess the porosity variation. Porosity varies depending on the distribution of the dust size and the shapes in the column prepared. This gives rise to the variation of porosity within 25% in the dust columns. The permeability (*K*) of the dust columns are measured incorporating permeability meter and *K* is estimated to be about 4 × 10^–16^ m^2^. The tests are repeated ten times using different dust columns prepared and findings revealed that the data for permeability (*K*) changes with 20%. This is attributed to different size and shapes of the dust particles in the dust column. Moreover, after considering the inertial force of the infusing fluid in the dust column is negligibly small as compared to capillary force^36^. The force balance between the force generated due to pressure drop and the force due to capillary pressure should be satisfied. This consideration yields the condition that the capillary force is same order of the force for pressure drop during the fluid infusion, i.e. $\pi {R}_{eff}^{2}\Delta {P}_{Cap}=2\pi {R}_{eff}{L}_{eff}{\Delta P}_{loss}$, where *L*_*eff*_ is the effective capillary length. The arrangement of the force balance leads to the equation for wetting fluid height, which becomes:

1 $$\frac{\mu \varepsilon }{K}h\frac{dh}{dt}+\rho gh=\frac{{\gamma }_{L}}{{L}_{eff}}$$

The effective capillary length is evaluated using the SEM micrograph (Fig. 5); however, the capillary length varies along the different locations of the dust column because of the variation of the dust size, dust shape, and the dust orientation in the dust column. Hence the approximate effective capillary length is considered as same as the column length (0.012 m). The solution of Eq. (1) yields^37^:

2 $$h=\frac{C \left[W\left(-exp\left[\frac{-({B}^{2}t+AC}{AC}\right]\right)+1\right]}{B}$$

The Lambert $W$ function has the series expansion, i.e.:

3 $$W\left(x\right)= \sum_{n-1}^{\infty }\frac{{(-1)}^{n-1} {n}^{n-2}}{(n-1)!} {x}^{n}$$

**Figure 6:**
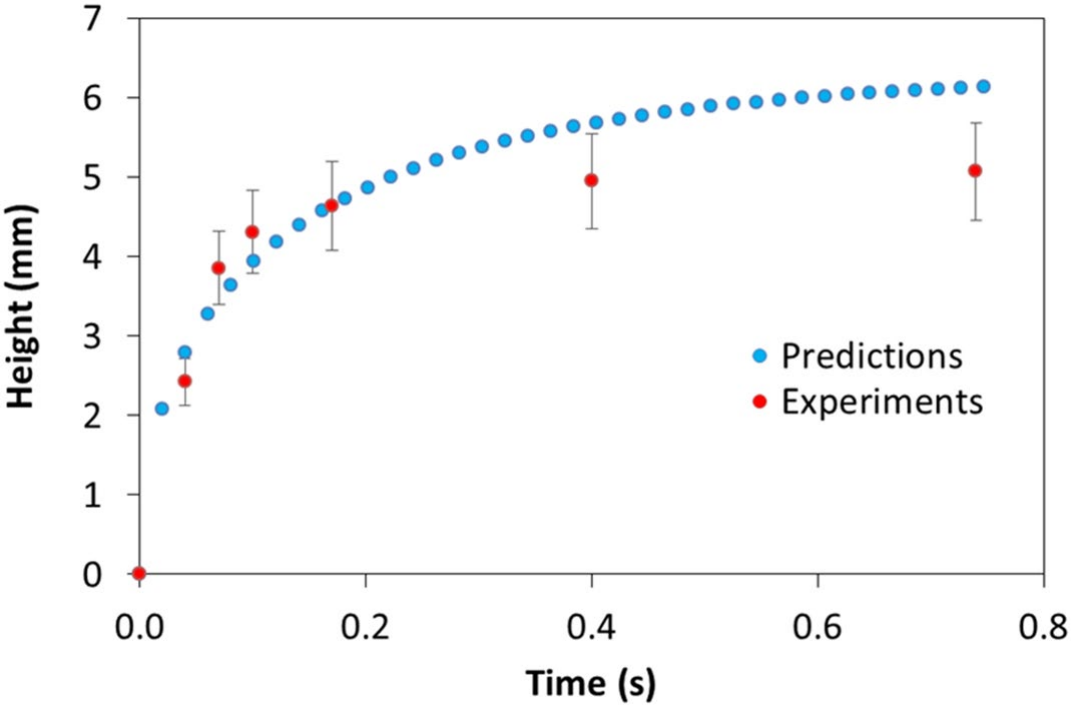
Wetting liquid height along dust column with time due to experiment and predictions of Eq. (1).

The Lambert function takes the form: $W\left(x\right)=x-{x}^{2}+ {\frac{3}{2}x}^{3}-{\frac{8}{3}x}^{4}+{\frac{125}{24}x}^{5}-{\frac{54}{5}x}^{6}+{\frac{16807}{720}x}^{7}+\dots$. The coefficients in Eq. (2) are: $A= \frac{\mu \varepsilon }{k}$, $B=\rho g$, and $C=\frac{{\gamma }_{L}}{{L}_{eff}}$ (Eq. 1). Incorporating the fluid viscosity, porosity, permeability, surface tension of fluid, and the effective capillary length, Eq. (2) can be solved and height (*h*) variation with time can be obtained. Figure 6 shows the wetting height (*h*) with time obtained from the experiment. The predicted wetting height is also included in Fig. 6. The wetting front of the liquid reaches 2.54 mm height in 0.04 s. Hence, the time for the droplet liquid infusing into the dust layer of 150 µm thickness on the hydrophobic surface becomes about 1 ms, which is less than the transition time of wetted length of the rolling droplet on the dusty surface (0.02 s). Consequently, the droplet fluid fully infuses and wets the dust layer on the hydrophobic surface during its rotational transition.

### Droplet motion on dusty surface

**Figure 7:**
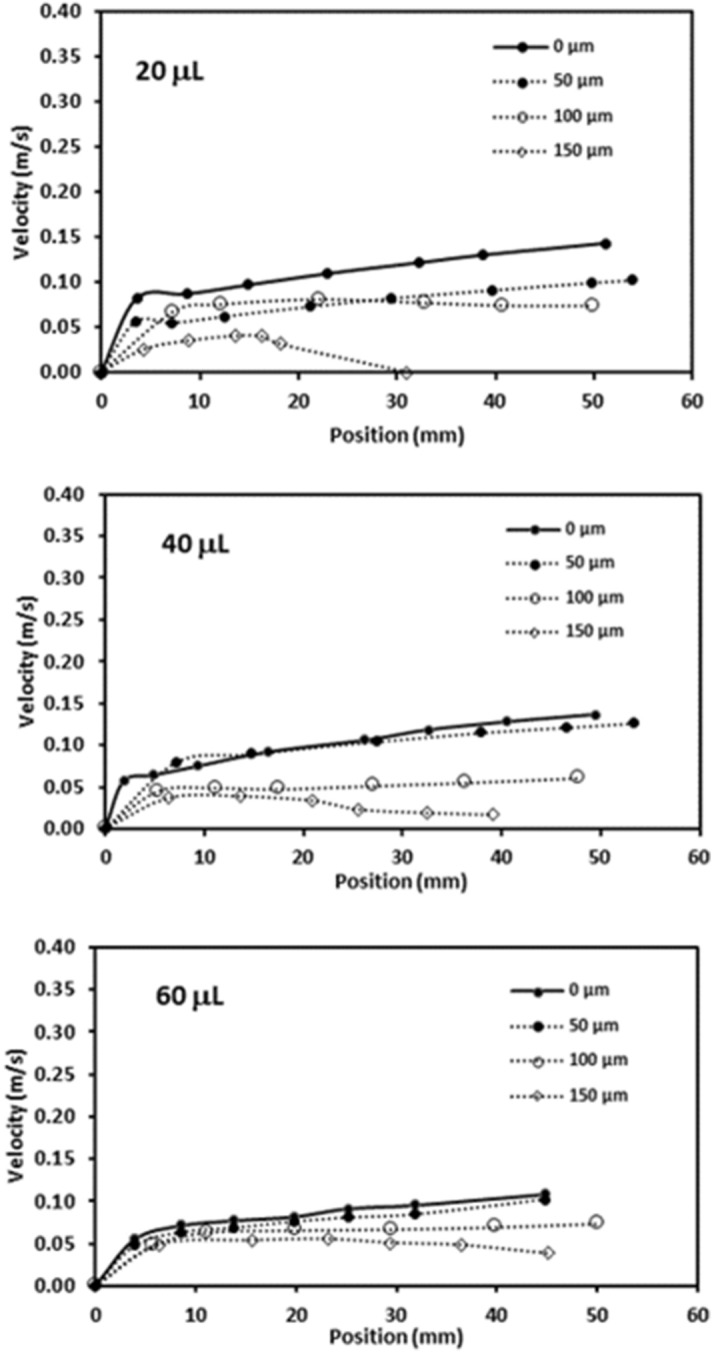
Translational velocity of droplet obtained from experiment on 1° inclined surface for various droplet volumes and dust layer thicknesses.

**Figure 8:**
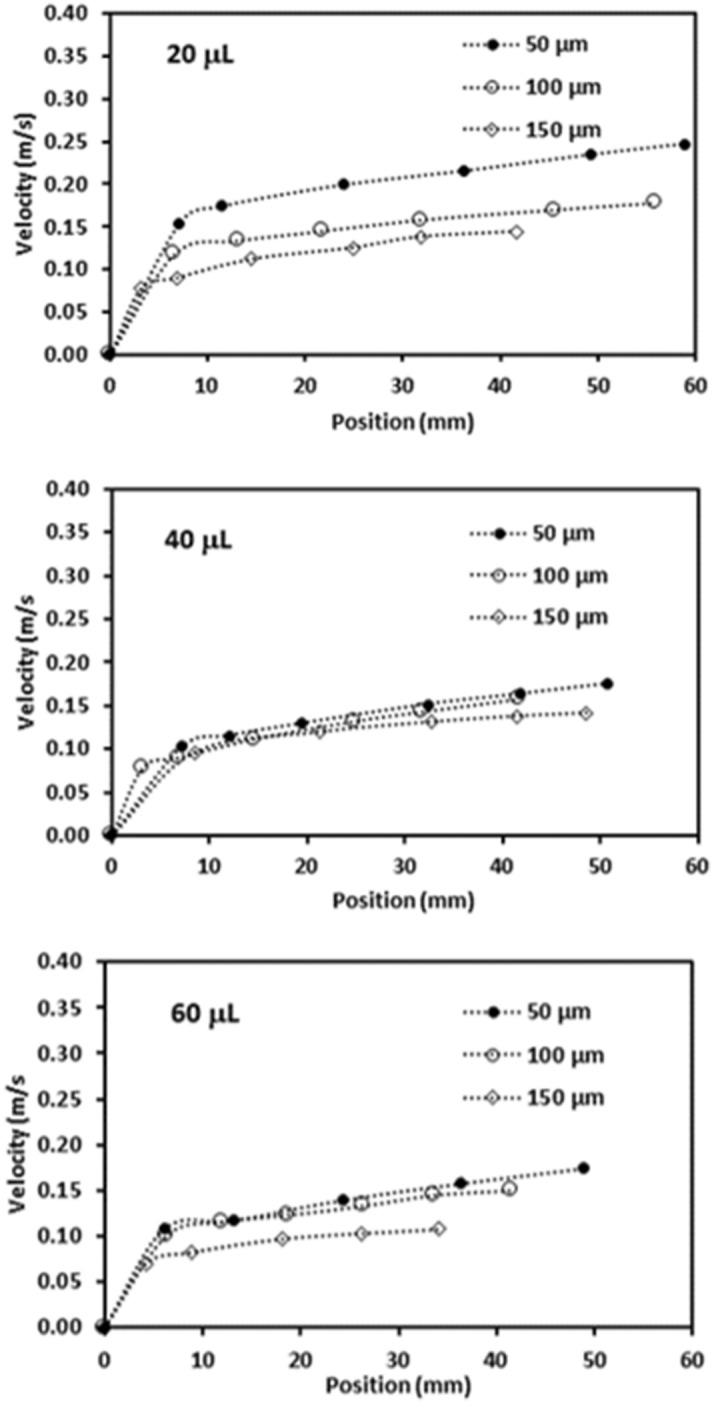
Translational velocity of droplet obtained from experiment on 5° inclined surface for various droplet volumes and dust layer thicknesses.

**Figure 9:**
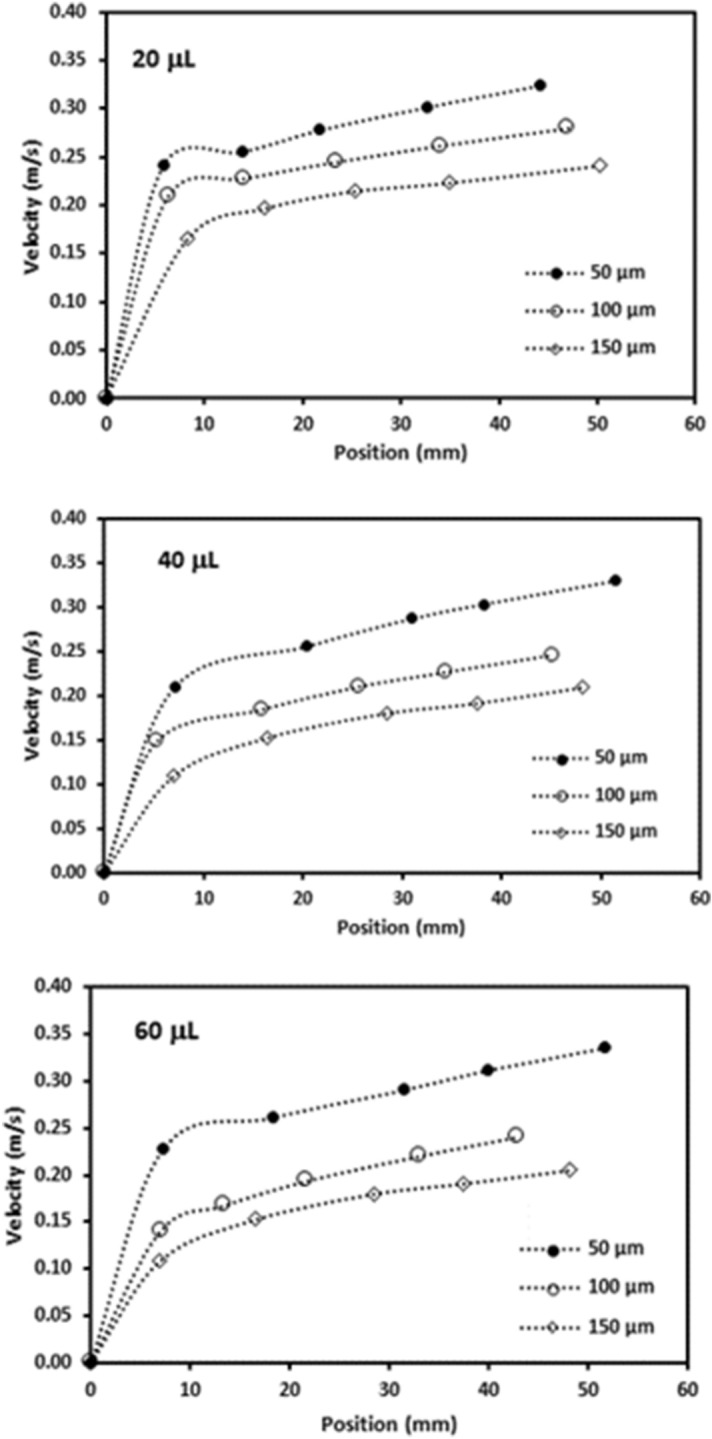
Translational velocity of droplet obtained from experiment on 10° inclined surface for various droplet volumes and dust layer thicknesses.

**Figure 10:**
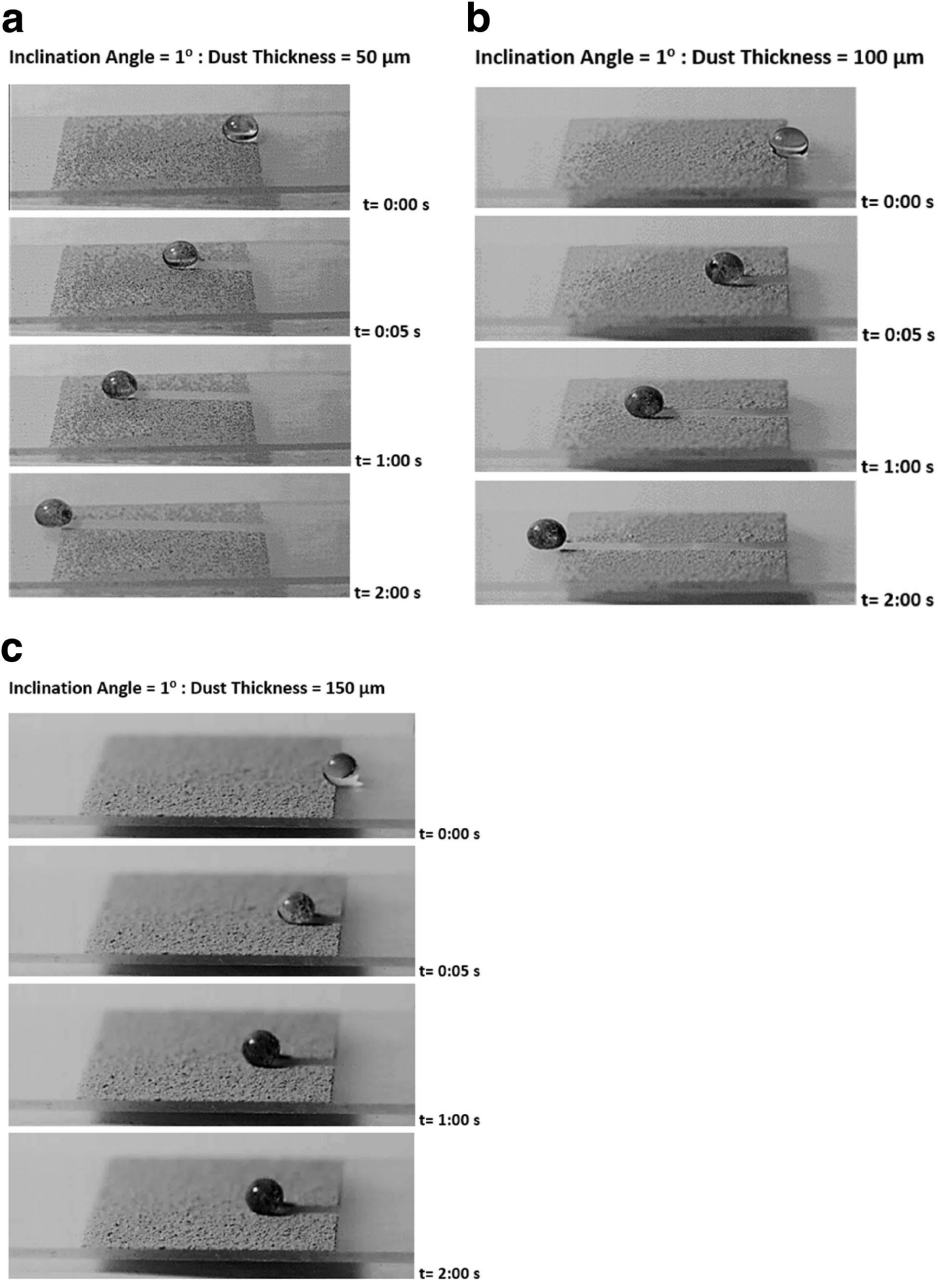
(**a**) Optical image of 20 µL droplet on dusty hydrophobic surface. Dust thickness is 50 µm. (**b**) Optical image of 20 µL droplet on dusty hydrophobic surface. Dust thickness is 100 µm. (**c**) Optical image of 20 µL droplet on dusty hydrophobic surface. Dust thickness is 150 µm.

**Figure 11:**
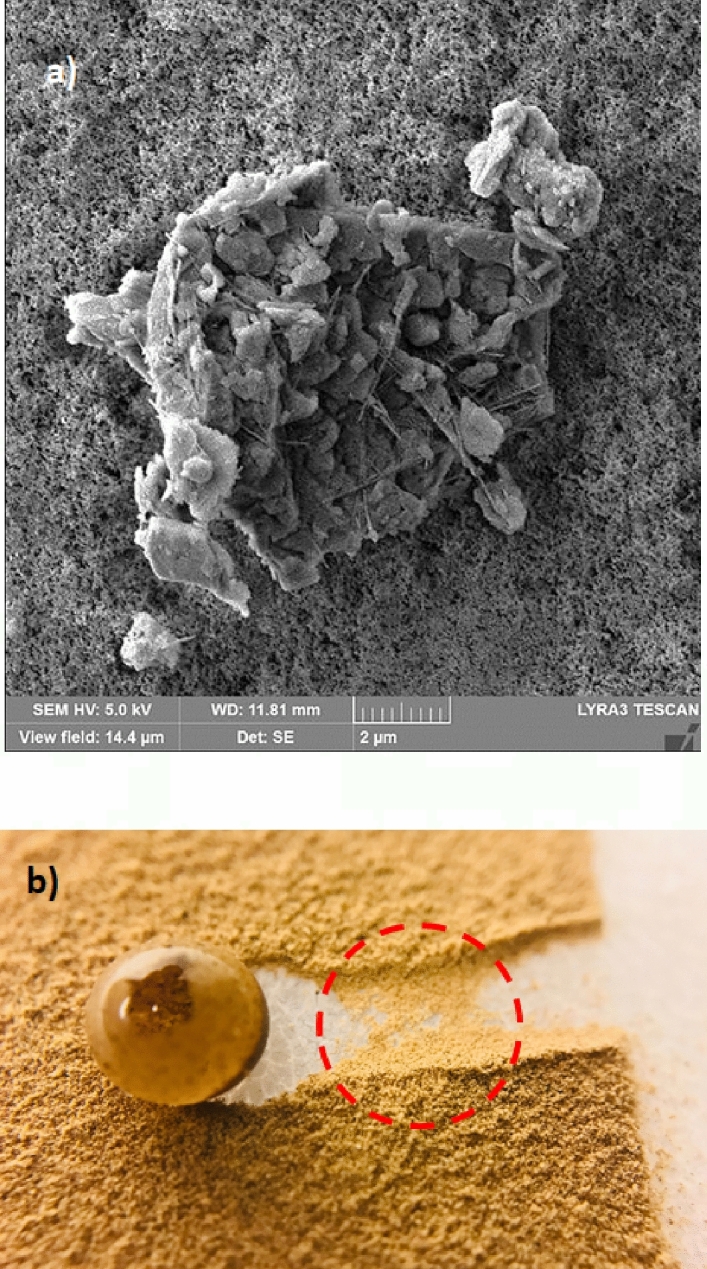
(**a**) SEM micrograph of dust residues on the coated surface. Dust particle has sharp edges, and (**b**) optical image of 20 µL droplet on 150 µm dust layer with inclination angle of 1° (droplet terminates on dusty surface). Circle shows dust residues on inclined dusty hydrophobic surface.

Figure 7 depicts translational velocity of the rolling droplet on the dusty surface for different dust thicknesses, 1° inclination angle of surface, and various droplet volumes. Translational velocity attains lower values for the dusty surface as compared to that corresponding to the clean hydrophobic surface with same wetting state. This is because of the resistance created between the droplet and the dusty surface, and dissolution of some dust compounds in droplet fluid. Dust compounds, such as KCl and NaCl, can dissolve in droplet fluid (water) causing increased surface tension^3^. Hence, experiments are carried out to determine change of droplet surface tension due to picking up of the dust particles. Surface tension of the droplet fluid increases from 0.072 to 0.119 mJ/m^2^ after mixing with the dust particles during rolling. Hence surface tension increase becomes almost 4%, which in turn enhances the pinning force (${F}_{pin}\cong \frac{24}{{\pi }^{3}}{\gamma }_{L}D{\phi }_{s}\left(cos{\theta }_{R}-cos{\theta }_{A}\right)$, where *D* is droplet wetting diameter, *ϕ*_*s*_ is solid fraction, *θ*_*R*_ and *θ*_*A*_ are receding and advancing angles of droplet^4^). However, the gravitational force (${F}_{grav}=mgsin\delta$, where *m* is droplet mass, *g* is gravitational acceleration, and *δ* is inclination angle of surface) remains larger than the pinning force^4^. Moreover, as the dust layer thickness increases, frictional and adhesion forces (causing pinning) lower the droplet velocity significantly, which is more pronounced for the large volume droplet (60 µL). This may be because of: (i) small inclination angle results in small gravitational force for rolling, and (ii) increasing droplet volume enhances wetting radius on the surface so that adhesion force increases. The droplet velocity reduces zero for 20 µL as the dust thickness becomes 150 µm. This is related to the inertial force acting on the droplet under the gravity, which becomes small as droplet mass reduces. Hence, the interfacial friction and adhesion force overcomes the inertial force generated under the gravitational pull. In addition, droplet velocity shows decreasing trend as the droplet volume and the dust thickness increase simultaneously. The balancing force for the droplet rolling is influenced by the increased wetting diameter and the dust thickness, i.e. enlarging wetting diameter due to droplet volume increase enhances the adhesion force; in addition, increasing the dust layer thickness boosts the frictional force. Figures 8 and 9 show droplet translational velocity with distance on the surfaces with dust presence for different droplet sizes and two inclination angles (5° and 10°). Increasing the dust thickness reduces the droplet translational velocity. In addition, increasing droplet volume lowers the translational velocity. This is attributed to large wetting diameter with increasing droplet size, which enhances the frictional and adhesion forces on the surface. As comparing Figs. 8 and 9, increasing inclination angle increases the droplet translational velocity because of the gravitational influence. This becomes more apparent for the small dust thicknesses. Hence, the droplet cleaning of the dusty hydrophobic surfaces is faster as the dust thickness becomes small. Figure 10a–c show high speed recording data for 40 µL droplet images on the dusty surface for various dust thicknesses. The droplet during its transition picks up the dust particles for all the dust thicknesses. As the dust thickness increases, amount of dust picked up by the droplet increases and visual transparency of the rolling droplet reduces. Large amount of dust mixing with droplet fluid occurs for the case of large dust thicknesses. This alters the droplet mass and increase the gravitational force acting on droplet; however, dissolution of some dust compounds (alkaline metal compounds) increases the liquid surface tension. Hence, the pinning force on the surface increases while creating adverse influence on the droplet translational velocity. Moreover, as the dust thickness increases, the ability of droplet picking up dust from the surface reduces. This gives rise to the dust residues remaining on the droplet path. The reduced ability of droplet picking up dust particles is related to (i) the amount of particles cloaked by the droplet fluid, during its transition, is limited and as the dust thickness increases, particles only cloaked by the droplet fluid are picked up by the rolling droplet, and (ii) short transition duration of the droplet wetted area on the dusty surface limits the cloaking and spreading of the droplet fluid on the surface of the dust particles. Figure 11a shows SEM micrograph of the dust residues on the surface. In addition, Fig. 11b depicts optical image of 20 µL droplet terminating on the thick dusty surface (150 µm thick) for the inclination angle of 1°. The dust residues have sharp edges and the residues with sharp edges can poke on the surface and they become hard to pick up by the rolling droplet. In the case of Fig. 11b, dusts are present on the droplet path and the point of droplet termination (end of transition on the surface), the droplet picks up almost all dust. The Bond number measures the importance of gravitational force over the surface tension force and it can be expressed as $Bo=\frac{\Delta \rho g{l}^{2}}{\gamma }$, here *Δρ* is the density variation in the droplet, *g* is gravitational acceleration, *l* is the droplet characteristics diameter, and *γ* is the surface tension. For small volume droplets (≤ 40 µL), the Bond number remains less than unity. For mall volume droplets, the wetting diameter on the surface is about 2.1–3.2 mm. However, for large volume droplet (60 µL), the Bond number is about 3.7, which is greater than unity and the wetting diameter on the surface is about 4.1 mm due to large puddling of the droplet. Hence, increasing Bond number causes large area of cleaning on the surface. However, mixing of the droplet fluid with dust particles slightly alters the surface tension, i.e. surface tension increases from 0.072 N/m to 0.119 N/m during rolling. This is because of dissolution of alkaline (Na, K) and alkaline earth (Ca) metals in the droplet fluid^3^. In addition, mixture of the droplet fluid and dust alters the droplet density from 1,000 kg/m^3^ to 1,070 kg/m^3^ during droplet rolling over the length scale of 5 mm on the dusty surface for dust layer thickness of 100 µm and droplet volume of 40 µL. This changes the Bond number slightly because change of density and surface tension as the droplet rolls on the dusty surface. Moreover, the change of surface tension is about 4% while the density increase is 7%. Hence, Bond number increases slightly as the droplet rolls over the surface. This is more pronounced with increasing droplet volume, i.e. amount of dust particles picked up increases.

## Conclusion

Cleaning of the dusty hydrophobic surface by the rolling water droplet is investigated and the effect of dust thickness on the dust removal is examined. Environmental dust is collected from local region (Dammam) in Saudi Arabia. The dust layer on the surface is considered to be porous wick structures and dust cloaking by the droplet fluid is analyzed. High speed video and optical imaging system is used monitoring the droplet motion on the inclined and dusty hydrophobic surface. To assess the effect of gravitational potential on dust picking by the droplet, experiments are repeated for different volumes of droplet and inclination angles of the dusty surface. To hydrophobize surface, functionalized nano-silica units are deposited on the plane glass samples using the dip coating method. In general, translational velocity of the droplet is influenced by the dust layer thickness. Increasing the dust layer thickness lowers the droplet velocity and for small droplet volume (20 µL), small inclination angle of the surface (1°), and large dust thickness (150 µm), the droplet motion terminates on the dusty surface. The specific findings of the present study are listed below:

- Coated surface has contact angle of about 150° ± 2° with hysteresis of about 2° ± 1°.
- Spreading of the droplet fluid on the dusty surface causes the cloaking of the dust particles. As the particles are cloaked by the fluid, they are picked up by the rolling droplet and the flow current developed in droplet interior carries these particles towards droplet interior.
- Increasing inclination angle of the dusty surface, enhances the droplet velocity and the droplet motion does not terminate on the dusty surface. Indicating that the droplet gravitational potential overcomes the pinning and the frictional forces during rolling.
- As the rolling droplet picks up dust, some dust compounds (alkaline metal compounds) dissolve in the droplet fluid while enhancing the surface tension by almost 4%. This increases the droplet pinning (adhesion) on the dusty surface. In the case of thick dust layer, the time taken for the droplet fluid wetting the dusty surfaces while penetrating into the dust layer becomes longer than the droplet wetting diameter transition over the dusty surface. This limits the amount of particles picked up by the rolling droplet. Hence, as the dust layer thickness increases, the dust residues on the surface becomes large. However, only few dust residues are observed on the surface covered by a thin dust layer (50 µm thickness). These residues are small and having the sharp edges, which anchor onto the hydrophobic surface.

Although detailed analysis for droplet rolling on a dusty hydrophobic surface and influence of dust layer thickness on droplet dynamics and dust removal mechanisms are presented, the outdoor testing of droplet rolling on dusty hydrophobic surfaces with various dust layer thicknesses is left for the future study. Nevertheless, the present study demonstrates the possibility of cleaning of the dusty inclined hydrophobic surface with various dust thicknesses by a rolling water droplet and gives in depth understanding of the mechanisms of the dust cloaking by the droplet fluid.
